# The *Streptomyces scabiei* Pathogenicity Factor Thaxtomin A Induces the Production of Phenolic Compounds in Potato Tubers

**DOI:** 10.3390/plants11233216

**Published:** 2022-11-24

**Authors:** Iauhenia Isayenka, Nathalie Beaudoin

**Affiliations:** Département de Biologie, Centre SÈVE, Université de Sherbrooke, Sherbrooke, QC J1K 2R1, Canada

**Keywords:** common scab, thaxtomin A, potato, phenolics, cell death

## Abstract

The phytotoxin thaxtomin A (TA) is the key pathogenicity factor synthesized by the bacteria *Streptomyces scabiei,* the main causal agent of common scab of potato (*Solanum tuberosum* L.). TA treatment of potato tuber flesh produces a brown color that was attributed to necrosis. The intensity of TA-induced browning was generally thought to correlate with potato sensitivity to the disease. In this study, we found that TA-induced browning was much more intense in the potato tuber flesh of the common scab moderately resistant variety Russet Burbank (RB) than that observed in tubers of the disease-susceptible variety Yukon Gold (YG). However, there was no significant difference in the level of TA-induced cell death detected in both varieties, suggesting that tubers response to TA does not correlate with the level of sensitivity to common scab. TA-treated potato tuber tissues accumulated significantly higher levels of phenolic compounds than untreated controls, with a higher phenol content detected in RB TA-treated tissues than in those of YG. Browning was associated with a significant induction of the expression of genes of the phenylpropanoid pathway in RB tubers, indicating that TA activated this metabolic pathway. These results suggest that tuber flesh browning induced by TA is due to the accumulation of phenolic compounds. These phenolics may play a role in the protection of potato tubers against *S. scabiei*.

## 1. Introduction

Common scab of potato causes tuber skin damage that reduces market value of tubers. While this disease mainly affects tuber skin by inducing corky lesions on the tuber surface, it can also affect deeper layers of the tuber, causing pitted lesions [1]. Common scab is frequently caused by the actinobacteria *S. scabiei* (syn. *S. scabies*), but infection with other pathogenic Streptomycetes species, such as *S. turgidiscabies* and *S. acidiscabies,* can also lead to this disease [1]. During infection, *S. scabiei* synthesizes the phytotoxin thaxtomin A (TA) which is the key factor for pathogenicity. TA production was shown to be essential for disease development since *S. scabiei* mutants impaired in the TA biosynthesis pathway could not infect plants [2,3,4]. TA has been shown to act as an inhibitor of cellulose biosynthesis, causing a variety of physiological effects on plant tissues and cells [5,6,7]. This toxin induced a rapid Ca^2+^ influx, caused cellular hypertrophy and activated an atypical programmed cell death in *Arabidopsis* cell cultures [8,9,10]. Recently, TA was identified as a chemical activator of the EDS1/PAD4 signalling that plays an important role in plant basal immunity against virulent biotrophic pathogens and in effector-triggered immunity [11]. TA was also shown to activate plant defense responses, such as the accumulation of the phytoalexin scopoletin in tobacco leaves and *Arabidopsis* seedlings and the synthesis of phenolic derivatives leading to ectopic lignification in etiolated *Arabidopsis* seedlings [12,13]. This variety of effects indicates that TA has an impact on various processes occurring in plant cells. However, it does not explain its specific mode of action.

TA has often been used for the preliminary screening of potato germplasm for resistance to common scab [14,15,16]. TA treatment of tuber slices induces tuber parenchyma browning, with an intensity that was proposed to correlate with sensitivity to common scab [14,17,18]. A few studies have reported a positive correlation between common scab disease resistance and toxin tolerance in potato shoots and tuber tissues [14,18,19]. In contrast, Tegg and Wilson (2010) [20] showed that the intensity of the browning response to TA was not associated with disease resistance when analyzing a collection of potato varieties with a known level of resistance to common scab. It was found that the RB potato variety, which is moderately resistant to common scab, was very sensitive to TA (causing intense browning), while the scab sensitive variety Tasman showed a high tolerance to TA (inducing light browning) [20].

In previous studies, TA-induced browning on tuber slices was attributed to cell death by necrosis [14,17,18,20,21]. However, it was suggested that morphological changes caused by pathogen infection and TA exposure on tubers were not characteristic of lesions resulting from necrotic cell death [22]. Browning of potato tubers due to various diseases and morphological changes can be caused by the accumulation of phenolic compounds in cells [23,24,25,26]. There is some evidence that synthesis of phenolics occurs during common scab infection. It was reported that TA treatment and TA habituation activate the expression of genes involved in the production of phenolic compounds leading to lignin and suberin synthesis [12,27]. In particular, TA treatment of *Arabidopsis* suspension cells upregulated gene expression of the defense-related phenylalanine-ammonia lyase (PAL) [8]. Both *S. scabiei* and TA were shown to stimulate the synthesis of the phenolic coumarin scopoletin [13]. In the common scab resistant plants regenerated from TA-selected cells, *S. scabiei* infection and TA induced the accumulation of suberin polyphenols in the periderm that was associated with the production of more phellem cell layers [28].

Based on these results, we hypothesized that the observed color change on TA-treated potato tuber flesh was not due to cell death but occurred as the result of the accumulation of phenol-containing substances. In this study, we observed that tubers from the moderately resistant to common scab RB variety appeared very sensitive to TA, developing large dark-brown areas after treatment with TA. In contrast, TA treatment induced small light brown areas in tubers of the common scab sensitive YG variety, similar to data published earlier in other susceptible varieties [20]. To test our hypothesis, the level of cell death induced by TA in tuber slices was estimated for both varieties using the cell viability dye Evans blue. Histochemical analysis of TA-treated tuber tissues was performed to evaluate morphological changes and phenolics accumulation in response to TA. We quantified phenol content in TA treated tissues and measured the expression of genes encoding enzymes of the phenylpropanoid pathway that leads to the synthesis of various phenolic compounds. Our results suggest that TA induced browning is associated with the accumulation of phenolic compounds.

## 2. Results

### 2.1. TA Induces Browning on Potato Tuber Slices

**Figure 1:**
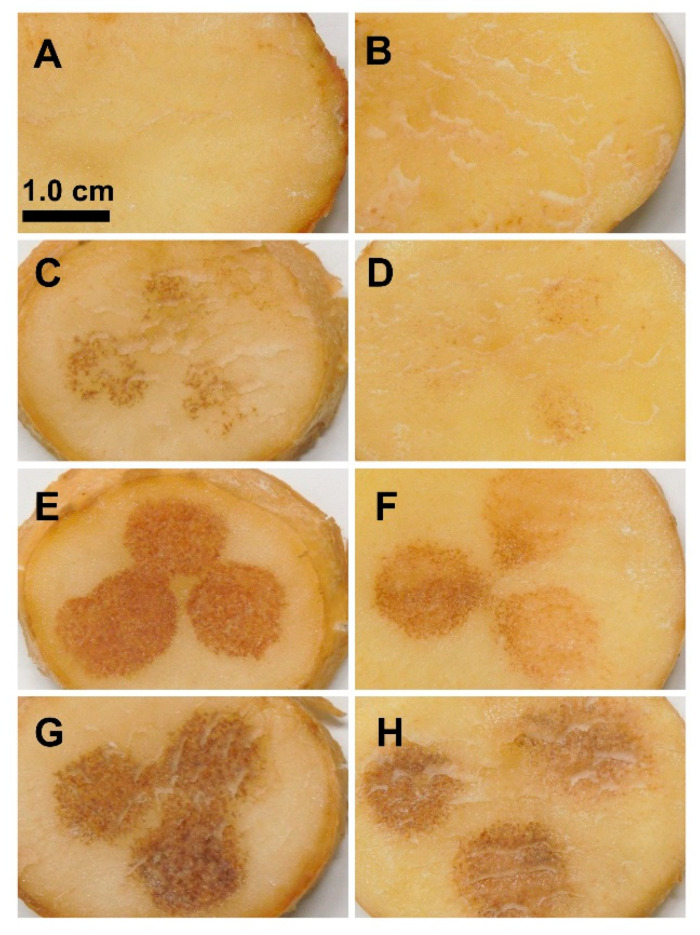
TA induces browning on RB (common scab moderately resistant) and YG (common scab sensitive) tuber slices. Representative pictures of tuber slices of RB (**A**,**C**,**E**,**G**) and YG (**B**,**D**,**F**,**H**) 6 days after treatment with methanol (MeOH) as control (**A**,**B**) and TA: 1 µM (**C**,**D**); 5 µM (**E**,**F**); 10 µM (**G**,**H**).

To evaluate TA’s effect on potato tubers, different concentrations of TA were applied on potato tuber slices of two varieties (RB and YG) with different sensitivity to common scab. Application of TA caused a brown coloration (browning) on the tuber slice surface for both varieties (Figure 1). The color intensity correlated with the concentration of the applied toxin for each variety. The lowest TA concentration (1 µM) was selected based on the induction of visible browning in RB tubers while the highest TA concentration was the one causing the most intense browning in YG tubers, i.e., 7 µM TA for small tubers (20–50 g) and 10 µM for large tubers (>75 g). However, browning was barely detected on slices of the scab-sensitive YG tubers treated with 1 µM TA compared to those of RB, a potato variety moderately resistant to common scab. At higher TA concentrations (5 and 10 µM), the brown coloration on YG tubers was still less pronounced than that observed in RB tubers. These results indicated that the level of resistance to common scab in these potato varieties was not consistent with the level of sensitivity to TA.

### 2.2. TA Induces Cell Death in Potato Tuber Parenchyma

**Figure 2:**
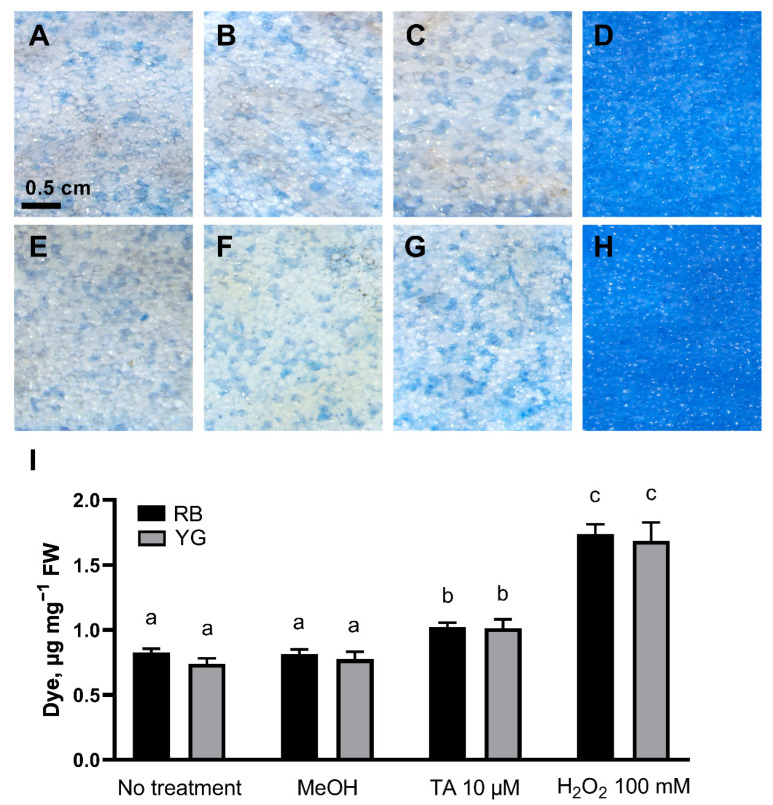
TA induces cell death in parenchymal cells of RB and YG tubers. Evans blue stained parenchyma sections of RB (**A**–**D**) and YG (**E**–**H**). (**A**,**E**) control conditions (no treatment); (**B**,**F**) sections treated with MeOH; (**C**,**G**) sections treated with 10 µM TA, (**D**,**H**) sections treated with H_2_O_2_ (100 mM) for 24 h. (**I**) Evans blue concentration (µg mg^−1^ FW) in tuber parenchymal sections untreated or after treatments with MeOH, TA (10 µM) or H_2_O_2_ (100 mM) for 24 h. Data is presented as the mean ± SEM of 6 biological replicates; letters (a, b, c) indicate significantly different means according to two-way ANOVA followed by Fisher’s LSD (*p* ≤ 0.05).

One of the main effects of exposing plant cells and tissues to TA is the induction of an atypical programmed cell death [8]. To determine whether tissue browning correlated with the induction of cell death by TA, the occurrence of cell death was evaluated in potato tuber flesh treated for 24 h with TA, hydrogen peroxide (as a positive control) and in control conditions (no treatment or treatment with methanol [MeOH]). Treated and control sections were incubated with the cell viability dye Evans blue, which is excluded from viable cells but specifically accumulates in dead cells by leaking through ruptured membrane [29,30] (Figure 2). The concentration of Evans blue extracted from tissues correlates with the level of cell death in these tissues. The concentration of Evans blue extracted from TA-treated RB and YG tuber sections was significantly higher (1.024 and 1.015 µg mg^−1^ fresh weight [FW]) than that obtained from non-treated (0.828 and 0.741 µg mg^−1^ FW) or MeOH-treated (0.816 and 0.776 µg mg^−1^ FW) tuber sections, showing that cell death in TA-treated tissues was significantly higher in parenchymal sections of both RB and YG tubers than in samples treated with MeOH or untreated controls. The amount of extracted dye from TA-treated sections did not differ significantly between the studied varieties (Figure 2I), indicating that TA caused a similar level of cell death in the potato tuber parenchymal cells of the RB and YG varieties.

### 2.3. TA Affects the Formation of the Wound Periderm

**Figure 3:**
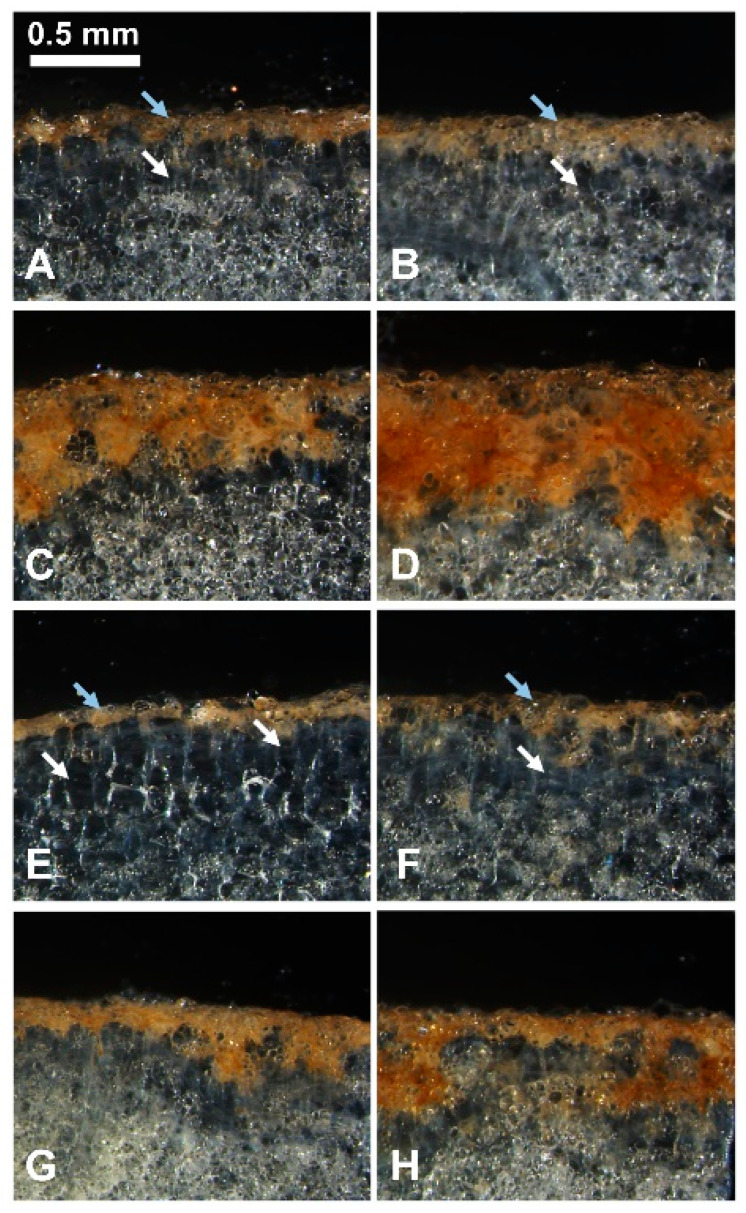
TA induces morphological changes in RB and YG tuber parenchyma. Photographs of sections cut perpendicular to the TA-treated surface of RB (**A**–**D**) and YG (**E**–**H**) tuber slices, 7 days after treatment. (**A**,**E**) no treatment; (**B**,**F**) treated with MeOH; (**C**,**G**) treated with 5 µM TA; (**D**,**H**) treated with 7 µM TA. White arrows show the phellogen formation, blue arrows point to the closing layer.

We performed histological examination of tuber flesh sites exposed to TA, MeOH or untreated control to clarify the cause of tissue browning (Figure 3). After seven days under control conditions, a slight darkening of the tissue was visible on the cut surface of the tuber section, occupying 1–2 layers of cells and representing a developed closing layer (Figure 3A,E, blue arrows). Rectangular cells that did not contain starch grains were visible under the closing layer. Presence of these cells indicates the beginning of phellogen formation, which is typical at this stage of wound periderm development (Figure 3A,E, white arrows) [31]. Application of TA caused browning in several layers of the tuber parenchyma cells lying under the closing layer in both RB (Figure 3C,D) and YG (Figure 3G,H). The browning reaction was more intense and spread out in the parenchyma tissues of RB (C and D) than those of YG (G and H). Higher concentrations of TA (7 µM) caused the formation of darker spots that went deeper in the parenchyma than for lower TA concentration (5 µM). While the structure and color of tuber cells treated with TA were similar to those of the closing layer cells, TA-treated cells showed altered forms and cellular hypertrophy.

### 2.4. Phenolic Compounds Deposition and Synthesis in Response to TA

**Figure 4:**
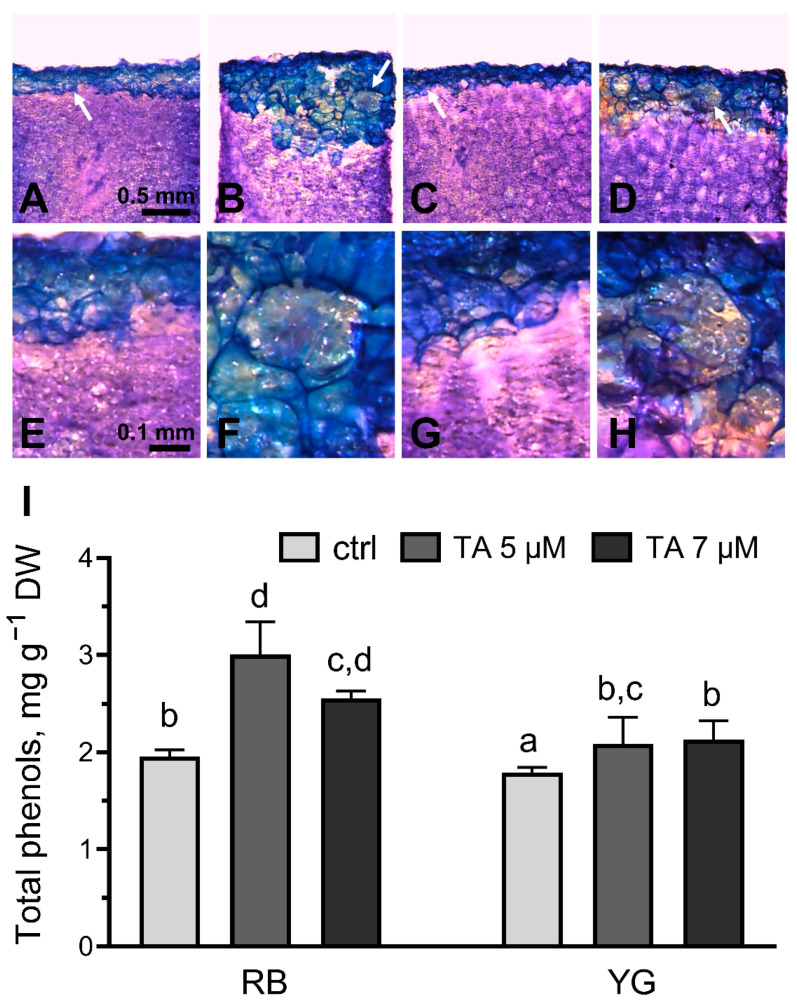
TA induces accumulation of phenolic compounds in tuber parenchyma. (**A**–**H**) Toluidine blue O staining of MeOH and TA (7 µM) treated tuber tissue, 9 days after treatment. (**A**–**D**) Phenolic deposits on the tuber slice surface after MeOH and TA treatment; (**E**–**H**) Close-up view of changes in cell morphology indicated by the white arrows in A-H; (**A**,**E**) RB slice treated with MeOH; (**B**,**F**) RB tuber slice treated with TA; (**C**,**G**) YG tuber slice treated with MeOH; (**D**,**H**) YG tuber slice treated with TA; (**I**) Total phenols (mg g^−1^ dry weight [DW] of tuber parenchyma tissue) extracted from the 5 µM and 7 µM TA-treated areas and control (ctrl) area of RB and YG tuber slices. Values are the means ± SEM (n = 3). Different letters (a–d) indicate significantly different means according to two-way ANOVA followed by Fisher’s LSD (*p* ≤ 0.05).

During the formation of the closing layer, the cell wall is strengthened by the accumulation of phenolic compounds [32]. When TA-treated tuber slices were stained with toluidine blue O, the area treated with TA was stained in blue, revealing the presence of polyphenolic compounds (Figure 4) [33]. The blue stained area was superposing the area of brown tissues. Under control conditions, blue-stained parenchyma cells were located strictly on the surface (Figure 4A,C), while after exposure to TA, the stained cells formed disordered masses both on the surface and in deeper layers (Figure 4B,D). The reaction to TA was more pronounced in RB tuber slices than those of the YG variety (Figure 4B,D). TA treatment also changed the morphology of tuber parenchyma cells. The shape of the cells located in the blue zone was altered, and some of the cells were swollen (or hypertrophied) (Figure 4E–H).

To confirm the accumulation of phenolic compounds in tissues treated with TA, total phenols were isolated from the TA-treated parenchymal regions of tuber slices, as well as the corresponding regions from control (MeOH-treated) slices (Figure 4I). TA treatment induced a significant difference in the content of phenolic compounds in RB and YG tuber tissues. As well, potato varieties RB and YG differed in the accumulation of phenolic compounds under TA treatment. Both studied factors were significantly different according to two-way ANOVA, with *p* values of 0.0191 and 0.0111, respectively. There was not statistically significant interaction between the varieties analysed and the effect of TA-treatment (*p* = 0.197). A higher concentration of phenols was found in control tuber tissues of the RB variety (1.96 mg g^−1^ DW) when compared to those from YG (1.79 mg g^−1^ DW). In RB tubers, treatment with 5 and 7 µM TA caused a significant accumulation of phenols up to 3.00 and 2.56 mg g^−1^ DW, respectively. In YG tubers, the accumulation of phenolic compounds reached 2.09 and 2.13 mg g^−1^ DW in 5 and 7 µM TA treated tubers, respectively. TA-induced accumulation of phenolic compounds was significantly higher in RB tubers than in YG tubers at TA concentrations of 5 and 7 µM.

The biosynthesis of polyphenolic compounds in plants occurs via the phenylpropanoid metabolic pathway. In the first steps of this pathway, the enzymes phenylalanine ammonia-lyase (PAL), cinnamate 4-hydroxylase (C4H), and hydroxycinnamoyl-coenzyme A shikimate:quinate hydroxycinnamoyl-transferase (HCT) convert phenylalanine into various intermediates, such as cinnamic, coumaric, caffeic and ferulic acids [34,35].

**Figure 5:**
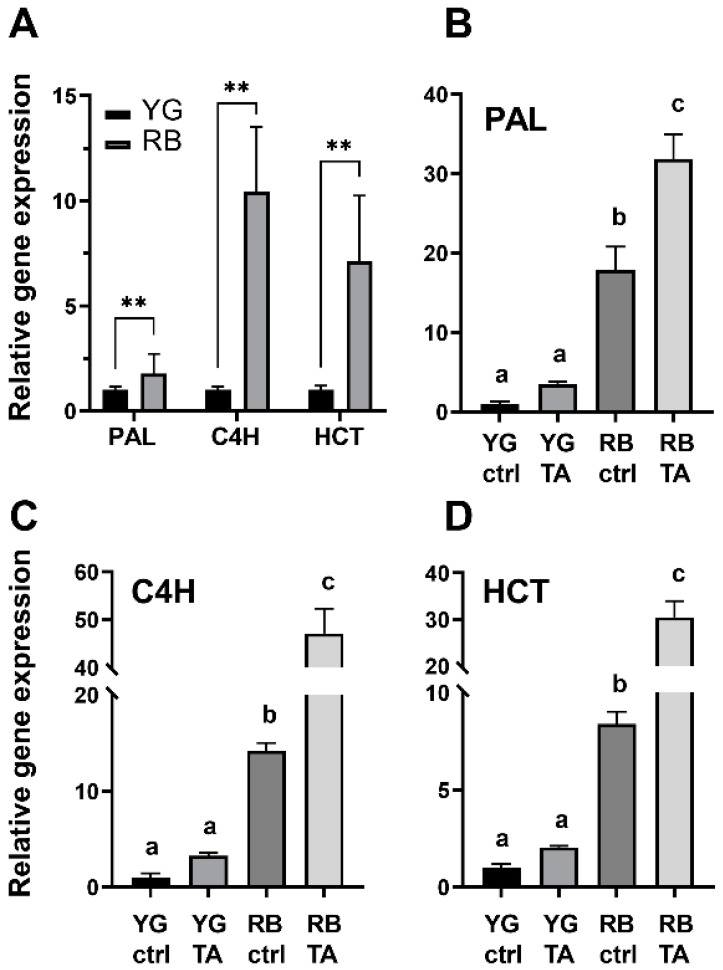
TA induces the expression of genes involved in phenylpropanoid biosynthesis in RB and YG tubers. (**A**) Relative gene expression of Phenylalanine ammonia-lyase (PAL), Cinnamate 4-hydroxylase (C4H) and Hydroxycinnamoyl-Coenzyme A shikimate:quinate hydroxycinnamoyl-transferase (HCT) in control (ctrl) mature potato tubers. Values represent means ± SEM (n = 4). The asterisks (**) indicate means statistically different according to two-way ANOVA followed by Fisher’s LSD (*p* ≤ 0.001). Relative gene expression of (**B**) PAL, (**C**) C4H, and (**D**) HCT 48 h after treatment with MeOH (ctrl) and TA (10 µM). qPCR data is presented as the mean of 4 biological replicates ± SEM; different letters (a, b, c) indicate significantly different means according to two-way ANOVA followed by Fisher’s LSD (*p* ≤ 0.05).

Using RT- qPCR analysis, we found that expression of the genes encoding enzymes of the phenylpropanoid pathway (PAL, C4H and HCT) was significantly higher in the tubers of the RB variety than in those of the YG variety (Figure 5). The relative expression in RB tubers compared to YG tubers showed a fold change of 1.78 for the PAL gene, 10.43 for the C4H gene and 7.11 for the HCT gene (Figure 5A). In potato slices treated with MeOH (control) for 2 days, expression of the PAL gene which encodes a key enzyme of the phenylpropanoid pathway, remained significantly higher (17.92-fold change) in RB tubers compared to the YG control (MeOH-treated) (Figure 5B). The expression of the genes encoding C4H and HCT enzymes in RB tubers had a 14.23- and 8.38-fold increase, respectively, compared to their expression in YG. In comparison to the YG control, TA induced a 3.50-fold increase in PAL gene expression in YG tuber slices, a 3.24-fold increase for C4H and a 2.05-fold increase for HCT. The highest level of gene expression was induced by TA in the tuber slices of the RB variety, with a fold increase of 31.88 for PAL, 47.13 for C4H and 30.51 for HCT when compared to the YG control. Two-way ANOVA showed a significant effect of genotype (RB and YG) on the expression of all genes tested (*p* < 0.0001), a significant TA-treatment effect for the gene expression of PAL (*p* = 0.0021), C4H and HCT (*p* < 0.0001), and a significant interactive effect of these variables on the gene expression of PAL (*p* = 0.019), C4H and HCT (*p* < 0.0001). These results show that expression of genes coding for enzymes of the phenylpropanoid pathway are differentially induced by TA in YG and RB tubers.

### 2.5. Inhibition of PAL Activity Reduces TA-Induced Browning

**Figure 6:**
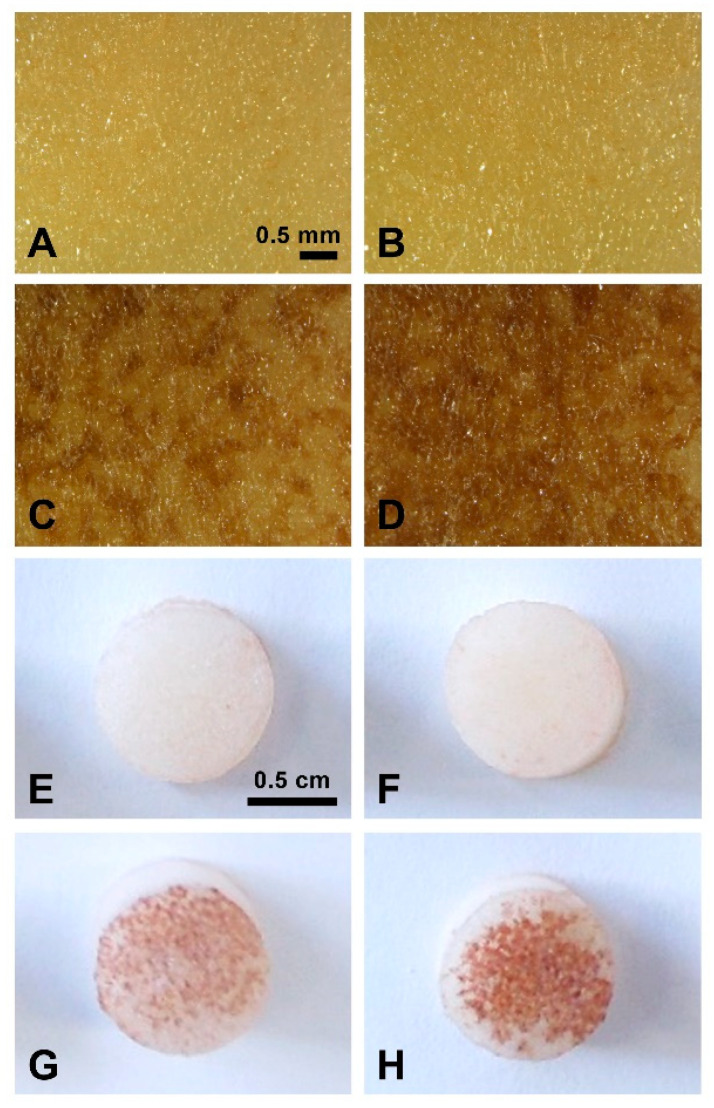
2-Aminoindan-2-phosphonic acid (AIP) inhibits tuber tissue browning in RB tuber parenchyma. Treatments were performed on tuber slices (**A**–**D**) or tuber discs (**E**–**H**). (**A**,**E**) Treatment with AIP (10 µM); (**B**,**F**) treatment with MeOH; (**C**,**G**) treatment with TA (5 µM) in combination with AIP (10 µM); (**D**,**H**) treatment with TA (5 µM). Pictures were taken 5 days after treatment and are representative of 3 tests.

The level of gene expression of enzymes of the phenylpropanoid biosynthesis pathway correlated with the degree of TA-induced browning on sections of tubers of RB and YG (Figure 1, Figure 3 and Figure 5). In particular, the expression of the PAL gene was increased by TA. PAL triggers a cascade of reactions that leads to the production of different classes of phenolic compounds, including phenols that can produce a brown coloration [36]. The PAL activity can be inhibited by the addition of the competitive inhibitor 2-aminoindan-2-phosphonic acid (AIP) [37]. Inhibition of the PAL enzyme by co-treatment of RB tuber slices with AIP and TA decreased the browning normally induced in TA-treated regions (Figure 6). These results suggest that inhibition of the phenylpropanoid pathway reduces the browning reaction induced by TA.

## 3. Discussion

Treatment of potato tuber slices with TA induces a dark brown coloration of the parenchyma tissue that is commonly thought to be due to necrosis (Figure 1) [14,17,18,21,38]. Since TA is the main pathogenicity factor of the common scab causative agent *S. scabiei*, this TA test has often been used to determine the level of sensitivity of different potato varieties to this disease [14,16,17,18]. However, our results indicated that the intensity of the browning response induced by TA did not correlate with the level of susceptibility to common scab resistance of the RB and YG potato varieties. (Figure 1). Tuber slices of common scab-moderately resistant variety RB appeared more sensitive to TA, showing a browning reaction that was more intense than that of YG, a common scab sensitive variety. Similar observations have been previously described for the common scab sensitive variety Tasman compared to RB, suggesting that the intensity of the browning induced by TA in plant tissues does not correlate with the level of resistance to common scab [20]. It was proposed that the induction of tissue darkening in response to TA depended on the genetic background of the potato variety [20]. We speculated that TA-induced browning was not the result of necrotic cell death caused by the toxin but would rather show a genotype-specific biochemical response to TA.

To test this hypothesis, we first evaluated the induction of cell death in TA-treated tubers using the cell viability dye Evans blue. While there is currently no direct evidence that the browning caused by TA on potato tuber slices is due to necrosis, TA has been shown to induce an atypical programmed cell death in *Arabidopsis* cell cultures that was different from necrosis [8]. Our results confirmed that the toxin increase cell death in the tuber parenchyma cells after 24 h (Figure 2). This cell death was associated with a characteristic swelling of cells, as previously described for TA-treated cell suspensions of *Arabidopsis* [6,7,8]. However, despite differences in the intensity of browning induced in RB and YG tubers, there was no significant difference in the level of cell death between both varieties. This indicates that while TA treatment rapidly induced cell death, the development of subsequent browning, which appeared from the third day after TA treatment, was not a symptom of cell death, but most likely a response to the toxin itself.

We further investigated the effect of TA in tuber tissues using histochemical analysis of tuber sections. Sectioning of potato tubers itself induces a wound healing response that stimulates the formation of a closing layer characterized by the induction of gene expression and suberization of the cell walls of parenchyma cells. This precedes the development of the wound periderm, a complex process characterized by the formation of well-defined tissues consisting of suberized phellem cells, cork cambium, and phelloderm that prevent tuber dehydration and pathogen infestation during wound healing [31,39]. Histological examination of tuber sections treated with TA revealed that the development of the closing layer and the wound periderm was altered. TA-induced browning was detected not only in the closing layer located on the surface, but also occurred in the underlying cell layers for both varieties (Figure 1 and Figure 3C,D,G,H). These changes were more pronounced in the moderately resistant variety RB than in the sensitive YG.

TA-induced browning was associated with the accumulation of phenolic compounds as revealed by Toluidine blue O dye staining (Figure 4A–H). Quantification of phenolics in control and TA-treated tissues confirmed that TA treatment significantly increased the accumulation of phenolics in both RB and YG tubers (Figure 4I). This was also correlated with the TA-induced expression of genes coding for enzymes involved in the first steps of the phenylpropanoid pathway, i.e., PAL, C4H and HCT, with a stronger induction detected in RB tubers compared to YG tubers (Figure 5). The accumulation of phenolics and induction of the phenylpropanoid in RB tubers in response to TA may explain the more intense browning detected in RB tuber tissues. Accordingly, inhibition of PAL activity, the first enzyme involved in the phenylpropanoid pathway, decreased TA-induced browning in tuber tissues (Figure 6). It was reported that the accumulation and oxidation of phenolic compounds produced by the chemical conversion of phenylalanine leads to the browning of plant tissues, particularly in the potato tuber parenchyma [40,41]. In particular, some 4-hydroxycinnamaldehydes synthesised via the phenylpropanoid pathway, such as *p*-coumaric and caffeic acid derivatives, are responsible for the development of yellow to brown color [36]. It is likely that some of these compounds are synthesized in response to TA, which would explain the darkening of tuber tissues detected after TA treatment.

Our results suggest that TA induced in tubers the formation of a phenol-containing protective layer that may be related to the wound periderm. Phenolic compounds synthesised in response to TA can be incorporated in the biopolymer suberin, which is a central component of the potato tuber periderm that forms the main barrier against pathogens [42,43]. Thickening of the tuber periderm and lenticel suberization have been reported in response to *S. scabiei* and TA, and in common scab resistant somaclones regenerated from TA-selected cells, suggesting that accumulation of phenolics may increase resistance to the disease [16,28]. Phenol accumulation was more important in the RB variety than in YG, suggesting that the strong induction of the phenylpropanoid pathway in RB tubers may contribute to producing a thicker suberin-enriched layer for tuber protection against *S. scabiei* [32,44,45]. The phenylpropanoid pathway is also involved in the production of phytochemical substances with antimicrobial properties, that play a role in protection against pathogenic organisms [13,39]. Several phenolic compounds have been shown to exhibit antifungal properties, helping the host plant to suppress pathogenic fungi [46]. In potato, it was reported that high chlorogenic acid content in tubers correlates with resistance to common scab [47]. A positive correlation was also shown between the content of phenolic acid in the skin of potato tubers and resistance to common scab [48]. Other phenylpropanoids, such as salicylic acid and phytoalexins, are key actors in the plant defense response and could also play a role in the protection against *S. scabiei* [49,50]. Lastly, we cannot exclude the possibility that phenolics that accumulated in tuber cells in response to TA may be implicated in a protective mechanism against cell death induced by TA. Parenchyma cell swelling was observed in cell layers located farther from the TA-treated tuber surface (Figure 3). Cell swelling, as well as cell death, are typical effects of TA’s action. Interestingly, TA-induced cell death can be alleviated by the use of auxin or auxin efflux inhibitors [51]. In *Arabidopsis* cell cultures, the accumulation of auxin in hypertrophied cells was suggested to maintain these cells alive. Several phenolic compounds are known to influence the polar transport of auxin [52]. Some of them, such as *cis*-cinnamic acid, exhibit auxin efflux inhibitor properties [53]. These data suggest that some phenolic compounds may protect tuber cells against TA-induced cell death.

## 4. Materials and Methods

### 4.1. Chemicals

All chemicals were purchased from Sigma-Aldrich Company unless otherwise indicated.

### 4.2. Plant Material

*In vitro* grown potato (*Solanum tuberosum* L.) plants, var. RB and YG were kindly provided by Les Semences Elite du Québec Inc. and Les Buisson Research Center Inc. (Pointe-aux-Outardes, QC, Canada). Potato plants were propagated on Murashige and Skoog (MS) medium supplemented with 3% sucrose (*w*/*v*), 0.7% agar (*w*/*v*) (BD Difco) and pH 5.7. Light conditions were set to 60–75 µmol m^−2^ s^−1^ with a light period of 16 h light/8 h dark cycle and constant temperature 20–22 °C in a Sanyo growth cabinet. Four-week-old plants propagated *in vitro* were planted in pots filled with soil mixture (soil:sand:vermiculite = 2:2:1) and grown at 22/18 °C under 16 h photoperiod with light intensity adjusted to 60–75 µmol m^−2^ s^−1^ in a Conviron plant growth chamber (PGR15). After one month, the photoperiod was changed to 12 h light/12 h dark cycle to stimulate tuber formation. Plants were irrigated twice a week and fertilized once a week with water soluble NPK (20:20:20) fertilizer. After 12 weeks of plant growth, tubers were harvested and used for analysis.

### 4.3. TA Extraction and Purification

Extraction and purification of TA produced from *S. scabiei* EF-35 [54] were performed as described previously [16]. Extracted TA was diluted in MeOH at a final concentration of 1 mM or 3.5 mM.

### 4.4. TA Test on Tuber Slices

Potato tubers were sterilised with 30% bleach solution in water for 20 min, dried under sterile laminar flow and aseptically cut into slices, as described previously [16,21]. Tuber slices were placed on sterile wet filter paper in Petri dishes. Discs of filter paper (5 mm) were soaked in MeOH as a control or in 1 µM, 5 µM and 10 µM of TA diluted in MeOH and dried under sterile air flow. The filters were transferred to the potato slice surface and a water drop (20 µL) was added to the top of the disc to ensure tight contact with the tuber surface. Tuber slices were stored in the dark at room temperature for 4 days unless otherwise specified.

### 4.5. Microscopy Analysis of TA-Induced Symptoms on Potato Parenchyma Cells

After conducting the TA test using 5 and 7 µM TA on potato tuber slices for 7 days (as described above), freehand sections cut perpendicular to the TA treated surface were prepared from tuber slices of RB and YG varieties. Light microscopy was performed on tuber sections using Leica stereomicroscope (M165FC).

### 4.6. Toluidine Blue O Staining of Phenolics Deposited in Potato Tuber Parenchyma Cells

Tuber flesh fresh hand-sections were incubated in bleaching solution consisting of 0.01% (*w*/*v*) Triton X-100 (Fisher Scientific) and 10% (*v*/*v*) commercial bleach for 24 h at room temperature to reduce pigmentation. Afterwards, sections were rinsed with distilled water and 100% ethanol [55]. Phenolics staining was performed by incubating tuber flesh sections for 10–15 s in Toluidine blue O 0.1% solution (*w*/*v*), a polychromatic dye that stains polyphenolic compounds in plant tissues in green to blue color [33]. Sections were rinsed with distilled water and observed with Leica stereomicroscope (M165FC) coupled with imaging system.

### 4.7. TA-Induced Cell Death Determination

The induction of cell death in potato tuber section was evaluated with a method modified from Vijayaraghavareddy et al., 2017 [30]. Six tubers of RB (tuber weight 80–90 g) and YG (75–90 g) were washed and surface sterilized with 30% bleach for 20 min. Sections of tuber flesh in the form of cylinders with a diameter of 10 mm were cut out aseptically with a cork borer. Each cylinder was split lengthwise with a scalpel into two parts and cut into thinner sections approximately 1 mm thick. Sections were incubated in liquid MS medium (pH 5.7) containing either 10 μM TA (57 µL 20 mL^−1^), the same volume of MeOH, 100 mM hydrogen peroxide or untreated (control) on an orbital shaker at 120 rpm for 24 h.

Parenchyma sections were rinsed with distilled water and stained in 5 mL of Evans blue dye solution (2.5 mg mL^−1^ Evans blue, CaCl_2_ 0.1 M, pH 5.6) for 20 min on an orbital shaker (120 rpm). After staining, sections were rinsed 3 times with distilled water. The excess water was removed from the sections with filter paper. Stained tuber parenchyma tissue (150 mg FW) was ground in 1 mL of a 1% SDS (*w*/*v*) (Fisher Scientific) solution using a mortar and pestle. After centrifugation, the optical density was measured at 600 nm using a microplate reader (SPARK multimode microplate reader, Tecan, Baldwin Park, CA, USA) and compared with an Evans blue standard curve. The standard curve was generated by plotting the absorbance (OD 600 nm) against the concentration of Evans blue standards (0.25 µg mL^−1^–2 mg mL^−1^).

### 4.8. Total Phenols Extraction and Determination

Slices of potato tubers were treated with 5 and 7 μM TA or MeOH (Fisher Scientific, Nepean ON, Canada) as described above for 5 days. The tuber flesh area treated with TA or MeOH was cut out of the slices and homogenised in liquid nitrogen. Samples were lyophilised for 3 days and stored at −80 °C until needed. Extraction of phenolic compounds was performed using lyophilised tuber flesh tissues as described previously [32]. Briefly, 100 mg of lyophilised tissue per sample were transferred to a glass tube. Each tissue sample was mixed with 1.54 mL of MeOH and 0.33 mL of water with 5 glass beads (2 mm) and 1 ceramic sphere (6.35 mm). The mixture was vortexed for 60 s before adding 0.77 mL of chloroform, and vortexed again for 15 min at room temperature. An additional 1.54 mL of chloroform-water (1:1 by volume) mixture was added to samples, after which the samples were sonicated for 15 min at room temperature and shaken at 120 rpm for another 15 min at room temperature. After centrifugation at 3000 rpm for 15 min at 4 °C, about 3.5 mL (containing both non-polar and polar soluble portion) were collected from the supernatant of the extraction mixtures with glass Pasteur pipettes. Extracted samples were kept in a liquid state at −20 °C before use. Total phenolics determination in samples was done using Folin–Ciocalteu reagent [41]. The concentration of phenols was determined by measuring optical density at 760 nm using gallic acid as a standard. Gallic acid concentrations from 0 to 400 μg mL^−1^ were used to generate a standard curve.

### 4.9. RNA Extraction and Gene Expression Analysis

RB and YG potato tubers (144–219 g) were surface sterilised with bleach for 30 min as described above and dried under a laminar flow hood. TA (10 µM) or MeOH (as a control) was applied under sterile conditions on 7 mm filter paper discs on tuber slices, as described above. TA-treated tuber slices were kept in the dark for 2 days. Tuber parenchyma samples treated with TA or MeOH were cut from the tuber slices with a cork borer. The upper TA- or MeOH-treated part of the cylindrical tissue sample (about 5 mm) was collected and combined with other samples of the same tuber. Samples were collected from 4 tubers, each tuber representing one biological replication. Samples were homogenised in liquid nitrogen with mortar and pestle and kept frozen at −80 °C.

RNA was extracted from harvested tuber parenchyma samples using phenol:chlorophorm method [56]. Samples were treated with TURBO DNAse (Invitrogen) according to supplier’s protocol. Two µg of extracted RNA were mixed with 1 µL of 0.5 µg/µL anchored oligo dT (IDT) in the final volume of 10 µL and heated at 70 °C for 5 min. After cooling down to 4 °C, 1.5 µL of AMVRT reverse transcriptase (Promega), 1 µL of RNAsin (Promega), 2.5 µL of dNTPs (10 mM; IDT) and 5 µL of 5x AMVRT buffer (Promega) were added to RNA samples to a final volume of 25 µL. cDNA synthesis was performed with the following conditions: 42 °C for 1 h, 70 °C for 10 min. Samples were cooled down to 4 °C, diluted 1:8 and kept frozen (−20 °C). For qPCR analysis, 1 µL of cDNA was mixed with 5 µL SYBR Green Master Mix (Bio-Rad) and 0.25 µL of each primer (F + R, 10 µM). RT-qPCR reaction was carried out using the following program: denaturation at 95 °C for 3 min (1 cycle), annealing at 95 °C for 15 s, 60 °C for 30 s (40 cycles), and extension at 95 °C for 1 min, 60 °C for 30 s, and 95 °C for 30 s (1 cycle) on QuantStudio 3 Real-Time PCR System (Thermo Fisher Scientific). Primers used in this study are listed in Supplementary Table S1 [57,58,59]. Potato 18S rRNA or adenine phosphoribosyltransferase 1-like (APRT) gene were used as internal control with comparable results [59]. The calculations of relative gene expression were made using the 2^−ΔΔCT^ method [60].

### 4.10. Inhibition of Phenylalanine Ammonia-Lyase Activity

Slices of RB tubers were treated with 5 μM TA as described above. For this assay, 20 μL of water or 20 μL of 10 μM AIP (SV ChemBioTech Inc., Edmonton, AB, Canada) was added to the filter disk just after its transfer to the surface of the tuber slice. Tuber slices were stored in the dark at room temperature. After 24 h, 20 µL of water were added to the discs. Tuber slices were kept in the dark at room temperature for 4 additional days. Color development on slices was observed and photo-documented with Leica stereomicroscope (M165FC) coupled with imaging system.

### 4.11. Statistical Analysis

Statistically significant (*p* ≤ 0.05) differences for TA-induced cell death, phenol quantification in RB and YG tissues, and relative gene expression in control and TA treated tissues were determined using two-way ANOVA followed by Fisher’s LSD. Statistical analysis and graphs were performed with GraphPad Prism software (v. 9.0).

## 5. Conclusions

TA-induced browning of potato tuber tissues was previously attributed to necrosis and thought to correlate with sensitivity to common scab. In contrast, our results indicate that TA-induced browning in tuber tissue primarily reflects the ability of a given potato variety to accumulate phenolic substances in response to TA, independently of cell death. We suggest that the accumulation of phenolics in TA-treated tuber tissues may have a protective role against pathogen infection by providing precursors for the synthesis of antimicrobial compounds or suberin, which is deposited on the tuber surface to form a protective barrier against pathogenic organisms.

This is the first time that the accumulation of phenolic compounds in response to TA is evaluated in potato tubers. This study sheds new light on the defense mechanism of tubers in response to the common scab-causing pathogen *S. scabiei* and may help further understand the mechanism of potato resistance to common scab.

## Data Availability

The data that support the findings of this study are available from the corresponding author on reasonable request.

## Supplementary Materials

The following supporting information can be downloaded at: https://www.mdpi.com/article/10.3390/plants11233216/s1, Table S1: List of primers.

## References

[1] Dees M.W., Wanner L.A. (2012). In search of better management of potato common scab. Potato Res..

[2] Goyer C., Vachon J., Beaulieu C. (1998). Pathogenicity of *Streptomyces scabies* mutants altered in thaxtomin A production. Phytopathology.

[3] Joshi M.V., Bignell D.R.D., Johnson E.G., Sparks J.P., Gibson D.M., Loria R. (2007). The AraC/XylS regulator TxtR modulates thaxtomin biosynthesis and virulence in *Streptomyces scabies*. Mol. Microbiol..

[4] Francis I.M., Jourdan S., Fanara S., Loria R., Rigali S. (2015). The cellobiose sensor CebR is the gatekeeper of *Streptomyces scabies* pathogenicity. mBio.

[5] King R.R., Lawrence C.H., Gray J.A. (2001). Herbicidal properties of the thaxtomin group of phytotoxins. J. Agric. Food Chem..

[6] Fry B.A., Loria R. (2002). Thaxtomin A: Evidence for a plant cell wall target. Physiol. Mol..

[7] Scheible W.-R., Fry B., Kochevenko A., Schindelasch D., Zimmerli L., Somerville S., Loria R., Somerville C.R. (2003). An *Arabidopsis* mutant resistant to thaxtomin A, a cellulose synthesis inhibitor from Streptomyces species. Plant Cell.

[8] Duval I., Brochu V., Simard M., Beaulieu C., Beaudoin N. (2005). Thaxtomin A induces programmed cell death in *Arabidopsis thaliana* suspension-cultured cells. Planta.

[9] Tegg R.S., Melian L., Wilson C.R., Shabala S. (2005). Plant cell growth and ion flux responses to the Streptomycete phytotoxin thaxtomin A: Calcium and hydrogen flux patterns revealed by the non-invasive MIFE technique. Plant Cell. Physiol..

[10] Errakhi R., Dauphin A., Meimoun P., Lehner A., Reboutier D., Vatsa P., Briand J., Madiona K., Rona J.P., Barakate M. (2008). An early Ca^2+^ influx is a prerequisite to thaxtomin A-induced cell death in *Arabidopsis thaliana* cells. J. Exp. Bot..

[11] Joglekar S., Suliman M., Bartsch M., Halder V., Maintz J., Bautor J., Zeier J., Parker J.E., Kombrink E. (2018). Chemical activation of EDS1/PAD4 signaling leading to pathogen resistance in *Arabidopsis*. Plant Cell Physiol..

[12] Bischoff V., Cookson S.J., Wu S., Scheible W.-R. (2009). Thaxtomin A affects CESA-complex density, expression of cell wall genes, cell wall composition, and causes ectopic lignification in *Arabidopsis thaliana* seedlings. J. Exp. Bot..

[13] Lerat S., Babana A.H., El Oirdi M., El Hadrami A., Daayf F., Beaudoin N., Bouarab K., Beaulieu C. (2009). *Streptomyces scabiei* and its toxin thaxtomin A induce scopoletin biosynthesis in tobacco and *Arabidopsis thaliana*. Plant Cell Rep..

[14] Wilson C.R., Tegg R.S., Wilson A.J., Luckman G.A., Eyles A., Yuan Z.Q., Hingston L.H., Conner A.J. (2010). Stable and extreme resistance to common scab of potato obtained through somatic cell selection. Phytopathology.

[15] Hiltunen L.H., Alanen M., Laakso I., Kangas A., Virtanen E., Valkonen J.P.T. (2011). Elimination of common scab sensitive progeny from a potato breeding population using thaxtomin A as a selective agent. Plant Pathol.

[16] Beaudoin N., Isayenka I., Ducharme A., Massie S., Gagnon A., Hogue R., Beaulieu C., Michaud D. (2021). Habituation to thaxtomin A increases resistance to common scab in ‘Russet Burbank’ potato. PLoS ONE.

[17] Tegg R.S., Gill W.M., Thompson H.K., Davies N.W., Ross J.J., Wilson C.R. (2008). Auxin-induced resistance to common scab disease of potato linked to inhibition of thaxtomin A toxicity. Plant Dis..

[18] Wilson C.R., Luckman G.A., Tegg R.S., Yuan Z.Q., Wilson A.J., Eyles A., Conner A.J. (2009). Enhanced resistance to common scab of potato through somatic cell selection in cv. Iwa with the phytotoxin thaxtomin A. Plant Pathol..

[19] Hiltunen L.H., Laakso I., Chobot V., Hakala K.S., Weckman A., Valkonen J.P.T. (2006). Influence of thaxtomins in different combinations and concentrations on growth of micropropagated potato shoot cultures. J. Agric. Food Chem..

[20] Tegg R.S., Wilson C.R. (2010). Relationship of resistance to common scab disease and tolerance to thaxtomin A toxicity within potato cultivars. Eur. J. Plant Pathol..

[21] Loria R., Bukhalid R.A., Creath R.A., Leiner R.H., Olivier M., Steffens J.C. (1995). Differential production of thaxtomins by pathogenic *Streptomyces* species in vitro. Phytopathology.

[22] Lawrence H.C., Clark M.C., King R.R. (1990). Induction of common scab symptoms in aseptically cultured potato tubers by the vivotoxin, thaxtomin. Phytopathology.

[23] Ampomah Y.A., Friend J. (1988). Insoluble phenolic compounds and resistance of potato tuber disc to *Phytophthora* and *Phoma*. Phytochemistry.

[24] Tomás-Barberán F.A., Espín J.C. (2001). Phenolic compounds and related enzymes as determinants of quality in fruits and vegetables. J. Sci. Food. Agric..

[25] Yadav V., Wang Z., Wei C., Amo A., Ahmed B., Yang X., Zhang X. (2020). Phenylpropanoid pathway engineering: An emerging approach towards plant defense. Pathogens.

[26] Liu X., Zhang A., Zhao J., Shang J., Zhu Z., Wu X., Zha D. (2021). Transcriptome profiling reveals potential genes involved in browning of fresh-cut eggplant (*Solanum melongena* L.). Sci. Rep..

[27] Brochu V., Girard-Martel M., Duval I., Lerat S., Grondin G., Domingue O., Beaulieu C., Beaudoin N. (2010). Habituation to thaxtomin A in hybrid poplar cell suspensions provides enhanced and durable resistance to inhibitors of cellulose synthesis. BMC Plant Biol..

[28] Thangavel T., Tegg R.S., Wilson C.R. (2016). Toughing it out—Disease-resistant potato mutants have enhanced tuber skin defenses. Phytopathology.

[29] Jacyn Baker C., Mock N.M. (1994). An improved method for monitoring cell death in cell suspension and leaf disc assays using Evans blue. Plant Cell Tiss. Organ Cult..

[30] Vijayaraghavareddy P., Adhinarayanreddy V., Vemanna R.S., Sreeman S., Makarla U. (2017). Quantification of membrane damage/cell death using Evan’s blue staining technique. Bio Protoc..

[31] Lulai E.C., Campbell L.G., Fugate K.K., McCue K.F. (2016). Biological differences that distinguish the 2 major stages of wound healing in potato tubers. Plant Signal. Behav..

[32] Jin L., Cai Q., Huang W., Dastmalchi K., Rigau J., Molinas M., Figueras M., Serra O., Stark R.E. (2018). Potato native and wound periderms are differently affected by down-regulation of FHT, a suberin feruloyl transferase. Phytochemistry.

[33] Schoenwaelder M.E.A., Clayton M.N. (1999). The presence of phenolic compounds in isolated cell walls of brown algae. Phycologia.

[34] Vogt T. (2010). Phenylpropanoid biosynthesis. Mol. Plant.

[35] Valiñas M.A., Lanteri M.L., ten Have A., Andreu A.B. (2015). Chlorogenic acid biosynthesis appears linked with suberin production in potato tuber (*Solanum tuberosum*). J. Agric. Food Chem..

[36] Liu S., Qi Q., Chao N., Hou J., Rao G., Xie J., Lu H., Jiang X., Gai Y. (2015). Overexpression of artificially fused bifunctional enzyme 4CL1–CCR: A method for production of secreted 4-hydroxycinnamaldehydes in *Escherichia coli*. Microb. Cell Fac..

[37] Jones A.M.P., Saxena P.K. (2013). Inhibition of phenylpropanoid biosynthesis in *Artemisia annua* L.: A novel approach to reduce oxidative browning in plant tissue culture. PLoS ONE.

[38] Loria R., Bukhalid R.A., Fry B.A., King R.R. (1997). Plant pathogenicity in the genus *Streptomyces*. Plant Dis..

[39] Sabba R.P., Lulai E.C. (2002). Histological analysis of the maturation of native and wound periderm in potato (*Solanum tuberosum* L.) tuber. Ann. Bot..

[40] Vitti M.C.D., Sasaki F.F., Miguel P., Kluge R.A., Moretti C.L. (2011). Activity of enzymes associated with the enzymatic browning of minimally processed potatoes. Braz. Arch. Biol. Technol..

[41] Teoh L.S., Lasekan O., Adzahan N.M., Hashim N. (2016). The effect of ultraviolet treatment on enzymatic activity and total phenolic content of minimally processed potato slices. J. Food Sci. Technol..

[42] Bernards M.A., Razem F.A. (2001). The poly(phenolic) domain of potato suberin: A non-lignin cell wall bio-polymer. Phytochemistry.

[43] Graça J. (2015). Suberin: The biopolyester at the frontier of plants. Front. Chem..

[44] Akyol H., Riciputi Y., Capanoglu E., Caboni M.F., Verardo V. (2016). Phenolic compounds in the potato and its byproducts: An overview. Int. J. Mol. Sci..

[45] Hammerschmidt R. (1984). Rapid deposition of lignin in potato tuber tissue as a response to fungi non-pathogenic on potato. Physiol. Plant Pathol..

[46] Lattanzio V., Cardinali A., Palmieri S. (1994). The role of phenolics in the postharvest physiology of fruits and vegetables: Browning reactions and fungal diseases. Ital. J. Food Sci..

[47] Johnson G., Schaal L.A. (1957). Accumulation of phenolic substances and ascorbic acid in potato tuber tissue upon injury and their possible role in disease resistance. Am. Potato J..

[48] Singhai P.K., Sarma B.K., Srivastava J.S. (2011). Phenolic acid content in potato peel determines natural infection of common scab caused by *Streptomyces* spp.. World J. Microbiol. Biotechnol..

[49] Dixon R., Paiva N. (1995). Stress-induced phenylpropanoid metabolism. Plant Cell.

[50] Dixon R.A., Lamb C.J., Masoud S., Sewalt V.J.H., Paiva N.L. (1996). Metabolic engineering: Prospects for crop improvement through the genetic manipulation of phenylpropanoid biosynthesis and defense responses—A review. Gene.

[51] Awwad F., Bertrand G., Grandbois M., Beaudoin N. (2019). Auxin protects *Arabidopsis thaliana* cell suspension cultures from programmed cell death induced by the cellulose biosynthesis inhibitors thaxtomin A and isoxaben. BMC Plant Biol..

[52] Cheynier V., Comte G., Davies K.M., Lattanzio V., Martens S. (2013). Plant phenolics: Recent advances on their biosynthesis, genetics, and ecophysiology. Plant Physiol. Biochem..

[53] Steenackers W., Klíma P., Quareshy M., Cesarino I., Kumpf R.P., Corneillie S., Araújo P., Viaene T., Goeminne G., Nowack M.K. (2017). *cis*-cinnamic acid is a novel, natural auxin efflux inhibitor that promotes lateral root formation. Plant Physiol..

[54] Faucher E., Savard T., Beaulieu C. (1992). Characterization of actinomycetes isolated from common scab lesions on potato tubers. Can. J. Plant Pathol..

[55] Beisson F., Li Y., Bonaventure G., Pollard M., Ohlrogge J.B. (2007). The acyltransferase GPAT5 is required for the synthesis of suberin in seed coat and root of *Arabidopsis*. Plant Cell.

[56] Kumar G.M., Iyer S., Knowles N.R. (2007). Extraction of RNA from fresh, frozen, and lyophilized tuber and root tissues. J. Agric. Food Chem..

[57] André C.M., Schafleitner R., Legay S., Lefèvre I., Aliaga C.A.A., Nomberto G., Hoffmann L., Hausman J.-F., Larondelle Y., Evers D. (2009). Gene expression changes related to the production of phenolic compounds in potato tubers grown under drought stress. Phytochemistry.

[58] Navarre D.A., Payyavula R.S., Shakya R., Knowles N.R., Pillai S.S. (2013). Changes in potato phenylpropanoid metabolism during tuber development. Plant Physiol. Biochem..

[59] Nicot N., Hausman J.F., Hoffmann L., Evers D. (2005). Housekeeping gene selection for real-time RT-PCR normalization in potato during biotic and abiotic stress. J. Exp. Bot..

[60] Livak K.J., Schmittgen T.D. (2001). Analysis of relative gene expression data using real-time quantitative PCR and the 2^−ΔΔCT^ method. Methods.

