# Supplementary material for: The Streptomyces scabiei Pathogenicity Factor Thaxtomin A Induces the Production of Phenolic Compounds in Potato Tubers
Source: Plants (Basel). 2022 Nov 24;11(23):3216. doi: 10.3390/plants11233216 (PMC9737112; doi:10.3390/plants11233216)
Supplement: Supplementary file 1 [file plants-11-03216-s001.zip › plants-2022095-supplementary.pdf]

Table S1. List of primers.

Gene	Primer sequence	Reference
Phenylalanine ammonia-lyase (PAL)	ACGGGTTGCCATCTAATCTGACA/ CGAGCAATAAGAAGCCATCGCAAT	[57]
Cinnamate 4-hydroxylase (C4H)	CCCAGTTTTTGGAAATTGGCTTCA/ GCCCCATTCTAAGCAAGAGAACATC	
Hydroxycinnamoyl-Coenzyme A shikimate: quinate hydroxycinnamoyl-transferase (HCT)	CCCGAATGCAGATACTGTTCCTGA/ AGTGAGTCCTCGTGCCATACAAGT	[58]
18S rRNA	AATTACCCAATCCTGACACGGG/ TTGCCCTCCAATGGATCCTCGTTA	[59]
Adenine phosphoribosyltransferase 1-like (APRT)	GAACCGGAGCAGGTGAAGAA/ GAAGCAATCCCAGCGATACG	
